# The Molecular Level Characterization of Biodegradable Polymers Originated from Polyethylene Using Non-Oxygenated Polyethylene Wax as a Carbon Source for Polyhydroxyalkanoate Production

**DOI:** 10.3390/bioengineering4030073

**Published:** 2017-08-28

**Authors:** Brian Johnston, Guozhan Jiang, David Hill, Grazyna Adamus, Iwona Kwiecień, Magdalena Zięba, Wanda Sikorska, Matthew Green, Marek Kowalczuk, Iza Radecka

**Affiliations:** 1 Wolverhampton School of Biology, Chemistry and Forensic Science, Faculty of Science and Engineering, University of Wolverhampton, Wolverhampton WV1 1LY, UK; Guozhan.jiang@wlv.ac.uk (G.J.); D.Hill@wlv.ac.uk (D.H.); M.Kowalczuk@wlv.ac.uk (M.K.); 2 Centre of Polymer and Carbon Materials, Polish Academy of Sciences, 41-800 Zabrze, Poland; Grazyna.Adamus@cmpw-pan.edu.pl (G.A.); ikwiecien@cmpw-pan.edu.pl (I.K.); mzieba@cmpw-pan.edu.pl (M.Z.); wsikorska@cmpw-pan.edu.pl (W.S.); 3 Recycling Technologies Ltd., South Marston Industrial Park, Swindon SN3 4WA, UK; Matt.Green@recyclingtechnologies.co.uk

**Keywords:** polyhydroxyalkanoates, PHAs, non-oxidized PE wax, N-PEW, *Cupriavidus necator* H16

## Abstract

There is an increasing demand for bio-based polymers that are developed from recycled materials. The production of biodegradable polymers can include bio-technological (utilizing microorganisms or enzymes) or chemical synthesis procedures. This report demonstrates the corroboration of the molecular structure of polyhydroxyalkanoates (PHAs) obtained by the conversion of waste polyethylene (PE) via non-oxygenated PE wax (N-PEW) as an additional carbon source for a bacterial species. The N-PEW, obtained from a PE pyrolysis reaction, has been found to be a beneficial carbon source for PHA production with *Cupriavidus necator* H16. The production of the N-PEW is an alternative to oxidized polyethylene wax (O-PEW) (that has been used as a carbon source previously) as it is less time consuming to manufacture and offers fewer industrial applications. A range of molecular structural analytical techniques were performed on the PHAs obtained; which included nuclear magnetic resonance (NMR) and electrospray ionisation tandem mass spectrometry (ESI-MS/MS). Our study showed that the PHA formed from N-PEW contained 3-hydroxybutyrate (HB) with 11 mol% of 3-hydroxyvalerate (HV) units.

## 1. Introduction

Polyhydroxyalkanoates (PHAs) are a family of polyhydroxyesters with 3-, 4-, 5-, and 6-hydroxyalkanoic acids that are biodegradable, non-toxic, biocompatible organic polyesters synthesized by some species of bacteria [1]. Currently there is a great deal of interest in PHAs due to their far-reaching applications, which include being used as biological implants, pharmacological delivery systems, packaging materials, and many more, as they can provide unique properties that do not exist in some synthetic polymers [1]. Traditional plastics, however, originate from petrochemical sources and have been a vital business commodity since their first industrial production. They are lightweight, cheap to manufacture, strong, flexible, and adaptable, but petro-based polymers form a growing proportion of the municipal solid waste (MSW) produced world-wide annually [2] and their waste materials are extremely harmful on a microscopic scale. These persistent plastics infiltrate the food chain as they are mechanically broken down (but not completely) in our oceans. Microplastics, defined as fragments that are less than five millimetres in diameter, can range from the size of an insect down to the size of a virus [3]. These particles, and larger plastics, also have a deadly impact on sea-life through entanglement or ingestion. It has been stated that plastics now account for somewhere between 60 and 80% of all ocean and shoreline debris [3,4,5,6]. The challenge, for both economic and environmental reasons, is to develop novel strategies that can create alternative materials to match or surpass the physical and commercial appeal of current petro-based plastic, with no adverse environmental consequences. 

Waste polyethylene (PE) is a potential carbon source that could be utilized to make value-added biopolymers, particularly as it is the most commonly-produced plastic, making up over 29% of worldwide plastic manufacture, while only 10% of it is recycled [7,8,9,10]. Unfortunately, the high molecular weight, hydrophobicity, and chemically-stable hydrocarbon covalent bonds of PE make it difficult for bacteria to metabolize effectively [11,12,13,14,15]. However, in the melt-form after oxidative degradation with free radical initiators and then sonication with an additional fermentation medium, PE waxes can be metabolized by bacteria and PHAs can be produced [16,17]. These polar PE waxes already have commercial uses; they can be used to produce PVC, as components of aqueous emulsions, lubricants, additives for varnishes, road marking paint, and adhesives [16,18,19,20]. The alternative non-polar, non-oxidized PE waxes (N-PEWs) have fewer industrial uses [7]. They are also cheaper and relatively easier to produce, which suggests that N-PEWs provide an alternative carbon source to O-PEWs. 

We propose that N-PEWs could be a pragmatic application of waste PE as a carbon source for PHA synthesis using *Cupriavidus necator* H16. The pyrolysis of PE samples in anaerobic conditions yields a complex mixture of relatively low molecular weight hydrocarbons with a molecular mass of 200–1000 [7,16]. Due to the hydrophobic nature of the non-oxidized waxes, ultrasound sonication can be performed to breakdown and distribute the wax as an emulsion so that it will be readily metabolized by bacteria [16,21]. For alkane degradation (with oxygen), the most widely-used pathway is the oxidation of the terminal methyl group into a carboxylic acid, via an alcohol intermediate, followed by complete mineralization through *β*-oxidation [15,22]. The intercellularly-synthesized PHAs are then held within bacterial species like *Cupriavidus necator* (formerly *Ralstonia eutropha* or *Alcaligenes eutropha)* as granules. *C. necator* is a Gram-negative, hydrogen-oxidizing bacterium (“knallgas”) that is able to grow at the interface of anaerobic and aerobic environments [12]. These microbes can synthesize PHA in the presence of excess amounts of carbon and limited nutrients [11] and they have been shown to metabolize a variety of carbon sources including fatty acids, hemicellulose, crude glycerol, methane, and even liquefied wood, for PHA production [11,23,24,25]. They are also able to accumulate up to 85% PHA per dry cell weight, often with 8–12 biopolymer granules per bacterium [16]. To extract the PHAs held within *C. necator*, their biomass is lyophilized and then hot solvent extraction is used, followed by precipitation in ethanol or hexane [16,23]. 

Here, we report on the production and molecular-level structural analysis of PHAs formed using *C. necator*, with non-oxidized wax as a carbon source in a nitrogen-rich tryptone soya broth (TSB) growth media. This is a novel use of N-PEWs with the aid of *C. necator*, which was selected due to its yield potential, limited resistance to copper metals (a possible contaminant from some wax production methods), growth rate at relatively low temperature, and its well-documented genetic profile and gene stability (for future modification and enhancement purposes) [16,25,26,27]. 

## 2. Materials and Methods

### 2.1. Microorganism

The bacterial species used for PHA production with N-PEWs and TSB or basal salts medium (BSM), was *C. necator* H16 (NCIMB 10442, ATCC 17699). This organism was obtained from the University of Wolverhampton stock culture (freeze-dried and kept at −20 °C). Previous to the study, cultures were revived and grown overnight at 30 °C (optimum) in TSB at 150 rpm. The microorganism was then sub-cultured on tryptone soya agar (TSA) plates and incubated at 30 °C for 24 h. 

### 2.2. Carbon Source and Chemicals

The non-oxidized wax was kindly provided by Recycling Technologies Ltd. (Swindon, UK) from waste plastics that were scanned and separated from contaminants (such as glass or stone) and then shredded and dried before chemical treatment [28]. These remnants were then passed to a thermal cracker, where the long hydrocarbon chains in the plastics were cracked into shorter chains. The hot hydrocarbon vapour leaving the reactor was then filtered and treated for impurities. At this stage the refined gas was condensed into a wax [28]. The wax was used as received without any further purification (more N-PEW structural details are found in Section 3.1). 

### 2.3. Media

TSB and TSA were purchased from Lab M Ltd. (Lancashire, UK). Both of these growth media were prepared under aseptic conditions, using the instructions of the manufacturer. The basal salts medium (BSM) used contained distilled water, 1 g/L K_2_HPO_4_, 1 g/L KH_2_PO_4_, 1 g/L KNO_3_, 1 g/L (NH_4_)_2_SO_4_, 0.1 g/L MgSO_4_·7H_2_O, 0.1 g/L NaCl, 10 mL/L trace elements solution. The trace element solution contained: 2 mg/L CaCl_2_, 2 mg/L CuSO_4_·5H_2_O, 2 mg/L MnSO_4_·5H_2_O, 2 mg/L ZnSO_4_·5H_2_O, 2 mg/L FeSO_4_, 2 mg/L (NH_4_)_6_Mo_7_O_24_·4H_2_O. BSM salts were purchased from BDH Chemicals Ltd. (Poole, UK). Ringer’s solution was also purchased from Lab M Ltd. (Lancashire, UK). A 1/4 strength tablet was used in 500 mL of deionized water; then it was completely dissolved. All media were sterilized by being autoclaved at standard conditions (121 °C for 15 min). 

### 2.4. Fermentation Procedure

Starter cultures were prepared using 20 mL TSB (in a 50 mL flask) inoculated with a single *C. necator* colony from a TSA spread plate. That culture was then incubated (aerobically) for 24 h at 30 °C and 150 rpm in a rotary incubator (Incu-Shake MIDI, Shropshire, UK). After 24 h these cultures were checked for contamination by Gram staining and microscope observation. 

Shake flask fermentation analysis was performed in triplicate using 500 mL wide neck Erlenmeyer flasks. Each flask used 1 g of N-PEW that was first put into a 50 mL beaker, covered with foil, and melted at 70 °C. Then 20 mL of sterile TSB or BSM was added to the melted wax, which caused the wax to become solid again. The temperature was then increased to re-melt the wax. The waxes were then sonicated (in TSB) for 8 min at 0.5 active and passive intervals with a power of 70% using a Bandelin Electronic sonicator, (Berlin, Germany) to form a TSB/wax emulsion or BSM/wax emulsion. This emulsion was tested for sterility by spread plating. The sterile emulsion was then added to 210 mL sterile TSB or BSM in a 500 mL flask, followed by 20 mL of the starter culture, giving a total volume of 250 mL. The experimental control was 230 mL TSB or BSM, inoculated with 20 mL of starter culture with no PE wax included. The flasks were incubated in a rotary incubator under the same conditions mentioned for 48 h.

Viable cell counts were done using the methodology of Miles and Misra [29]. Briefly, 5 mL samples were aseptically collected from the flask cultures at 0, 3, 24, and 48 h. Serial dilutions of each sample were performed to 10^−8^ and then 20 µL of each dilution was pipetted onto a standard TSA plate in triplicate. These plates were incubated at 30 °C for 48 h and the colonies were counted and expressed in log_10_ CFU mL^−1^.

### 2.5. PHA Extraction Procedure

PHA extraction was performed after the 48 h fermentation time period had elapsed. The flasks were removed from their incubators and the flask contents were filtered using a sieve (to remove any conjugates of wax) and separated into centrifuge tubes, usually 35–40 mL per tube. The media was then centrifuged in a Sigma 6-16KS centrifuge for 10 min at 4500 rpm. At this point, there was only the pellet containing the biomass and the emulsion layer visible. The supernatant in each tube was discarded and the biomass was obtained and frozen overnight a −20 °C. This was followed by lyophilization using an Edwards freeze-drier (Modulyo, Crawley, UK) for 48 h at a temperature of −40 °C and at a pressure of 5 MBAR. The dry biomass was then weighed and recorded as cell dry weight (CDW) before being placed into an extraction thimble and, using Soxhlet extraction with HPLC grade chloroform for 48 h, the PHA was collected as a chloroform/biopolymer mixture. Then rotation evaporation was used (at 50 °C) to remove the chloroform. Polymer precipitation using n-hexane was performed in a round-bottom flask and then this was further separated by filtration (Watman No. 1 paper). If required, hexane was used to rinse the product further to remove any residue low molecular weight wax. The sample was then left in a fume cupboard to dry for up to five hours and the yield was recorded using:

Percentage yield of PHA = (weight of extracted polymer)/(cell dry weight) × 100

### 2.6. Characterization

#### 2.6.1. FTIR 

Fourier transform infrared spectroscopy (FTIR) was performed using a DuraScope (Genesis II) spectrophotometer (Smiths Detection Inc., Danbury, CT, USA). After a background scan, samples of N-PEW were placed under the lens. FTIR spectra were recorded in the transmittance mode with a resolution of 1 cm^−1^ in the range of 4000–400 cm^−1^.

#### 2.6.2. GPC

Gel permeation chromatography (GPC) was used to find the molecular weight and molecular weight distribution of the wax and polymers produced during this study. The analysis was conducted using a TOSOH EcoSec HLC/GPC 8320 (Tosoh Bioscience, Yamaguchi, Japan) system equipped with a RI and a UV detector (Tosoh Bioscience, Yamaguchi, Japan) operating at a temperature of 40 °C. The column used was TSKgel HZM-N (Tosoh Bioscience, Yamaguchi, Japan), calibrated against polystyrene standards. The UV detector was set at a wavelength of 254 nm. Chloroform was used as the eluent, at a flow rate of 0.25 mL/min. A sample size of 2 μL was injected into the system using an autosampler.

The wax GPC analysis included: (i) wax pre-sonication; (ii) post-sonication; (iii) post-fermentation (wax and TSB) and the control; (iv) post-shake flask N-PEW in TSB. The number-average molar mass (Mn) and the molecular mass distribution index (Mw/Mn) of the N-PEWs were determined in a CHCl_3_ solution at 35–60 °C heating to melt the waxes, then 10 min of cooling, to ascertain whether the waxes were fully dissolved in the selected volume of HPLC-grade chloroform. This N-PEW/chloroform solution was carefully pipetted into a syringe and passed through a filter into a GPC ampoule. 

#### 2.6.3. NMR Analysis

Proton nuclear magnetic resonance (^1^H-NMR) spectra were recorded with a Bruker Avance II (Bruker, Rheinstetten, Germany) operating at 600 MHz, with 64 scans, 2.65 s acquisition time, and an 11 µs pulse width. ^13^C-NMR spectra were recorded with a Bruker Avance II operating at 150.9 MHz, with 20,480 scans, 0.9088 s acquisition times, and 9.40 µs pulse width. The ^1^H-NMR and ^13^C-NMR spectra were run in CDCl_3_ at room temperature with tetramethylsilane (TMS) as an internal standard. 

#### 2.6.4. ESI-MS/MS Analysis and Identification of PHA at the Molecular Level

Electrospray mass spectrometry analysis was performed using a Finnigan LCQ ion trap mass spectrometer (Thermo Finnigan LCQ Fleet, San Jose, CA, USA). The oligomer samples, prepared as described by Kawalec et al. [30], were dissolved in a chloroform/methanol system (1:1 *v*/*v*), and the solutions were introduced into the ESI source by continuous infusion using the instrument syringe pump at a rate of 5 μL/min. The LCQ ESI source was operated at 4.5 kV, and the capillary heater was set to 200 °C; the nebulizing gas applied was nitrogen. For ESI-MS/MS experiments, the ions of interest were isolated monoisotopically in the ion trap and were activated by collisions. The helium damping gas that was present in the mass analyser acted as a collision gas. The analysis was performed in the positive-ion mode. 

PHA with low molar mass was obtained via thermal degradation of bacterial PHA in the presence of potassium hydrogen carbonate (KHCO_3_). The respective amount of polyester and salt, at a ratio PHA/KHCO_3_ equal to 5, was introduced in a vial with ethanol. The mixture was then stirred for 30 min to provide a homogeneous suspension. The experiment was carried out in an oven at 180 °C. The length of time for the process was dependent on the molar mass plain PHA. For the PHAs with a mass bellow 100,000 it was 20 min, and for polymers with a mass between 200,000 and 400,000, it was 30 min. This time regime allowed for the obtaining of oligomers with a molar mass of around 2000. After the experiment, oligomers were protonated with the Dowex^®^ 50WX4 in hydrogen form. The oligomer and Dowex^®^ (1:1 wt%) was dissolved in chloroform and the mixture was stirred for four hours. Next, the Dowex^®^ was removed by filtration. The oligomers obtained were characterized by GPC and ^1^H-NMR spectrometry, as well as their structure at a molecular level using ESI-MS/MS. The ^1^H-NMR analysis revealed the presence of characteristic signals corresponding to the protons of 3-hydroxybutyrate (HB) (and 3-hydroxyvalerate (HV)) repeating units, and also the signals attributed to the crotonate end groups. 

## 3. Results

### 3.1. N-PEW Initial FTIR and NMR Analysis

**Figure 1:**
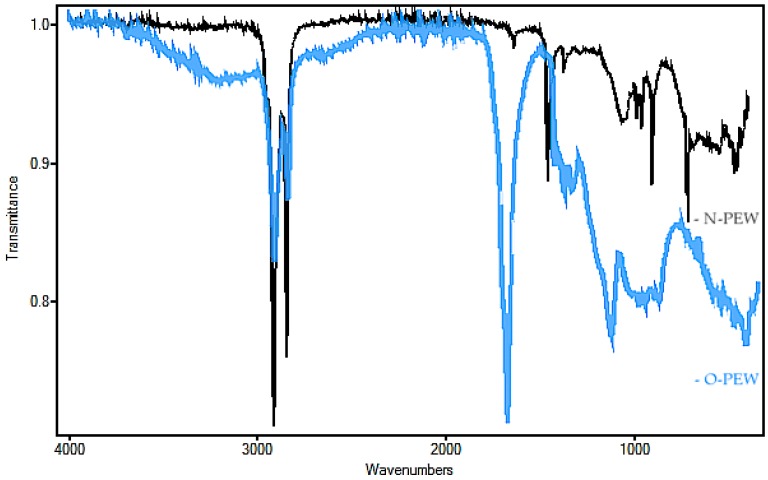
FTIR of O-PEW (blue trace) and N-PEW (black trace) used in this study.

The non-oxidized wax obtained for this study was tested to confirm its structure and chemical properties. The waxes were produced from waste plastic underwent thermal cracking to produce a refined gas that was then condensed into a wax [28]. FTIR was performed on O-PEW and N-PEW for comparison and to determine the presence or absence of functional groups associated with oxidation (Figure 1). 

The N-PEW (black) spectrum showed a strong absorption at 2800 cm^−1^, an indication of a C-H stretch, which confirms saturated chains. There was no absorbance at 3500 cm^−1^ (C=O stretch overtone) and weak absorbance at 1790–1700 cm^−1^ (C=O stretch), as compared with the spectrum of the oxidized wax, further confirmation of hydrocarbon chains with a very small degree of oxidation. When these results are compared to the O-PEW (blue) reading (a wax with an acid number of 197), there was less absorbance at 2800 cm^−1^ than what is displayed in the N-PEW reading. There was also a greater absorbance at 1700 cm^−1^ and broad absorbencies at 1160–1060 cm^−1^ which are likely due to R-O-R aliphatic bonds.

**Figure 2:**
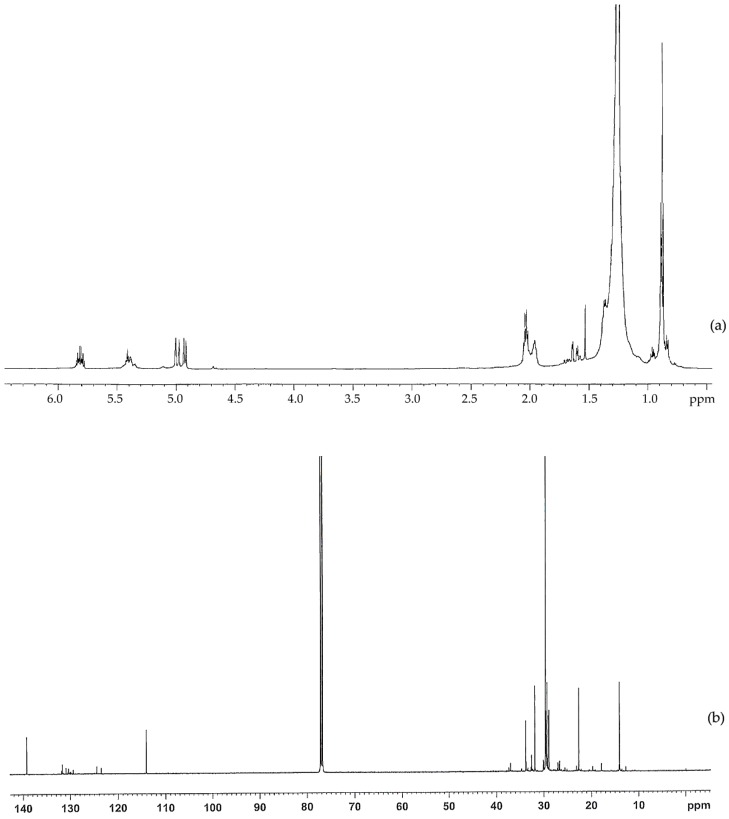
(**a**) ^1^H-NMR and (**b**) ^13^C-NMR spectra for the non-oxidized polyethylene wax used in this study.

The structure of the non-oxidized PE wax was further studied using ^1^H-NMR and ^13^C-NMR, as shown in Figure 2a,b. In the ^1^H-NMR, there are strong signals at 0.88 and 1.25 ppm, which are attributed to CH_3_ and CH_2_. The signals at 1.6 and 2.0 ppm may be due to the allylic hydrogen or hydrogen adjacent to the C=O group. There are signals at 4.9 ppm, 5.4 ppm, and 5.8 ppm in the ^1^H spectrum, and there are signals at 114 ppm and 139 ppm in the ^13^C spectrum.

These signals indicate that there are very small amounts of oxygenated functional groups in the non-oxidized wax. However, the amount of oxygenated functional groups are much less than those in the oxidized wax.

### 3.2. Growth of Bacteria and PHA Yield

**Figure 3:**
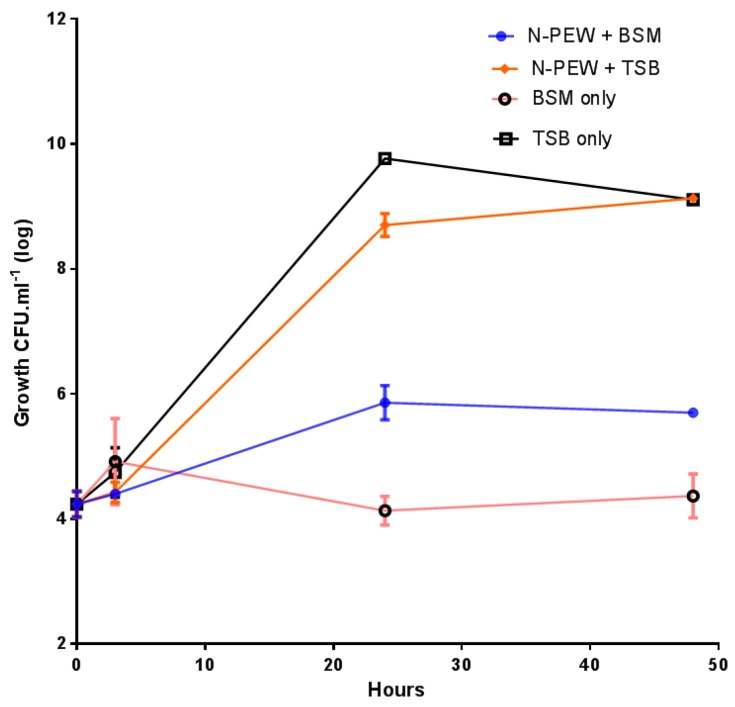
Small-scale fermentations of *C. necator* H16 with 4 g/L of N-PEW in TSB or BSM. Data points are arithmetic means of triplicates, while error bars denote the SE of the mean.

The growth of *C. necator* H16 in TSB only (control), BSM only, and TSB/BSM supplemented with N-PEW is shown in Figure 3.

The bacteria were inoculated at approximately 4.1 log_10_ CFU mL^−1^ and grew to 9.1 log_10_ CFU mL^−1^ with TSB only and TSB with N-PEW as an additional carbon source. There was no significant difference (*p* < 0.5) between these growth curves, however, when the growth curves with and without N-PEW and BSM are examined, it is evident that there is a difference in final counts. There was little growth when BSM only was used, cell numbers were merely maintained at 4.1–4.3 log_10_ CFU mL^−1^) over 48 h, but when N-PEW was added to BSM, there was more growth, an increase of 1.8 log_10_ CFU mL^−1^, and the counts ended at 5.8 log_10_ CFU mL^−1^ rather than 4.3 log_10_ CFU mL^−1^ in BSM only. In addition, there was no product yield with BSM only and with BSM with N-PEW, suggesting that the wax, alone, is not sufficient for PHA production, but it can sustain cells (Table 1). When TSB only and TSB with N-PEW were used the product was visually different; the PHA produced from TSB only was paper-like in texture and white, while the PHA produced from the N-PEW with TSB was more brittle. 

### 3.3. GPC of PHA and N-PEW 

GPC analysis of the PHAs obtained using N-PEW and TSB as growth medium showed that the number-average molar mass (Mn) was in the range of 64,000 g/mol with a molecular distribution of 9.7 (Mw/Mn). The Mn results for polyesters synthesized using TSB only were lower, in the range of 52,000 g/mol with a distribution of 5.7 (Mw/Mn).

GPC analysis of the waxes under different conditions during this investigation is reported in Table 2. The N-PEW results showed the number-average molar mass of normal N-PEWs used was (Mn) was 1600 g/mol with a weight average (Mw) of 5400 g/mol.

The GPC results showed that before sonication there were two distinct types of N-PEW in the sample: high molecular weight (the first peak) and lower molecular weight chains (second peak). After the sonication procedure (Section 2.4) the higher molecular weight peak was removed as the high molecular weight chains were broken down to a Mn of 1200 g/mol with a molar mass index of 1.2. However, any further reduction in the Mn value cannot be attributed to bacterial activity as the flasks without bacteria produced similar Mn values of 1100 g/mol after 48 h.

### 3.4. PHA Identification and Characterization

**Figure 4:**
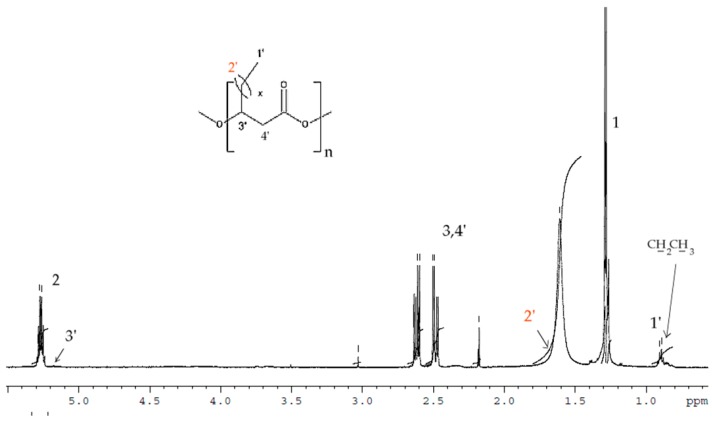
^1^H-NMR spectra of PHAs produced from small-scale fermentations with *C. necator* H16 using TSB media supplemented with N-PEW as a carbon source.

The ^1^H-NMR results of the PHAs formed from shake-flask fermentation experiments with TSB and N-PEW using *C. necator* is shown in Figure 4.

In the spectrum the signals corresponded to the protons of the HB repeating units (labelled 1, 2, and 3) and additional signals at δ = 0.9 (-CH_2_CH_3_) and δ = 5.17 (-CH) correspond to HV-repeated units that were observed. After integrating these signals, the content of monomer units other than 3-hydroxybutyrate (3-HB) has been estimated at 11 mol% 3-hydroxyvalerate (HV). Due to some of overlapping of signals, the protons of 2′ are not visible. 

### 3.5. ESI-MS/MS

**Scheme 1:**
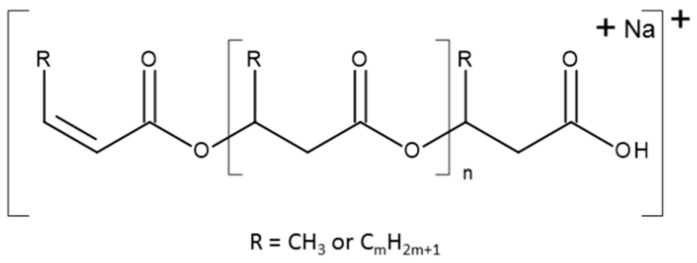
The general formula of ions observed in the ESI-MS spectra.

**Figure 5:**
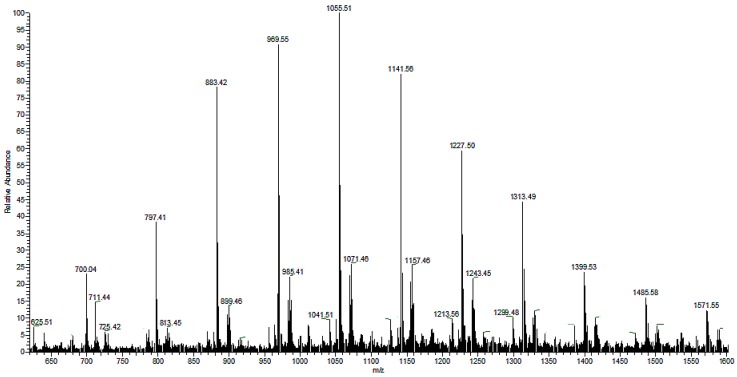
The ESI-MS spectrum (positive-ion mode) of the PHA oligomers, obtained via partial thermal degradation of the biopolyester produced by *C. necator* H16 utilizing TSB and N-PEW as an additional carbon source.

**Figure 6:**
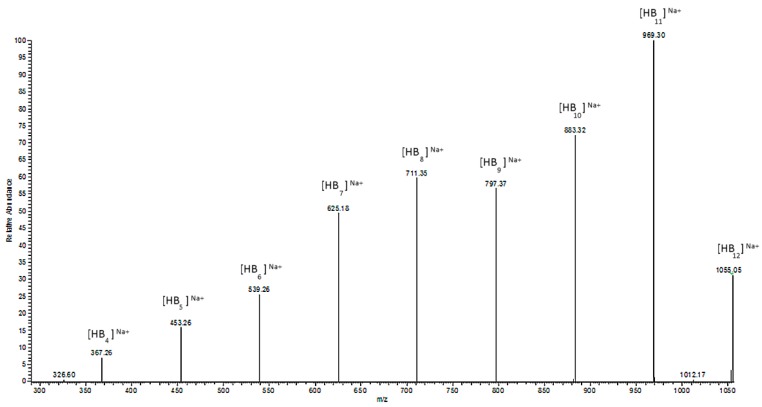
ESI-MS/MS spectrum for the selected sodium adduct ion of oligomers of [HB_12_ + Na]^+^ at 1055 *m*/*z*.

**Figure 7:**
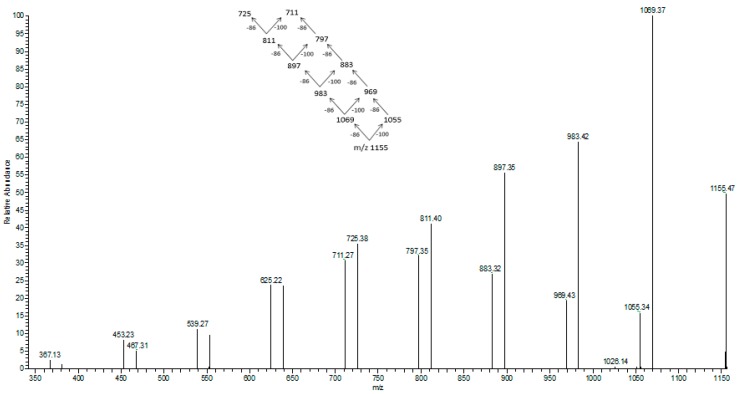
ESI-MS/MS spectrum obtained for the selected sodium adduct of oligomers of [HB_13_HV + Na]^+^ at *m*/*z* at 1155 *m*/*z*.

The controlled thermal degradation of PHAs synthesized from N-PEW, induced by KHCO_3_, was performed according to the procedure referred to in Section 2.6.4 [30]. This kind of E1cB degradation leads to PHA oligomers with unsaturated and carboxylic end groups. The results of PHAs formed from TSB with N-PEW are displayed in Figure 5, Figure 6 and Figure 7. Scheme 1 shows the general structure of the ions presented in the ESI-MS spectra.

There are two series of ions visible in the mass spectrum. The main series of the ions at *m/z* 1571, 1485, 1399, 1313, 1277, 1141, 1055, 969, 883, and 797 corresponds to the sodium adduct of oligomers with crotonate and carboxyl end groups, calculated according to the formula *m*/*z* 86 + (86 × n) + 23, as shown in Scheme 1. There are also a small series of ions besides the main series. The *m*/*z* difference is 14 between the main series and the small series, which corresponds to a CH_2_ group.

The ESI-MS^2^ spectrum of the sodium adduct of the biopolyester oligomers at *m*/*z* 1055 is presented in Figure 6. The product ions at *m*/*z* 969, 883, 797, 711, 625, 539, 453, and 367 correspond to the oligo (3-hydroxybutyrate) terminated with carboxyl end groups. 

In Figure 7 the fragmentation of the sodium adduct ion of oligomer of [HB_13_HV + Na]^+^ at *m*/*z* at 1155 *m*/*z* is presented. This parent ion produces fragment ions of subsequent oligomers according to the theoretical fragmentation pathway diagram. The loss of 86 Da equates to the loss of crotonic acid (e.g., 1155–1069 *m*/*z*), and the loss of 100 Da equates to an HV unit. The fragmentation spectrum of these ions confirms that the most intensive ions in the clusters correspond to sodium adducts of 3-hydroxybutyrate oligomers. Therefore, the fragmentation spectrum for the precursor ion at *m*/*z* 1155 confirms the presence of 3-hydroxybutyrate and randomly-distributed 3-hydroxyvalerate co-monomer units in polyester chains produced from N-PEW and TSB.

## 4. Discussion

The FTIR spectra showed that the N-PEWs received were non-polar, saturated hydrocarbons (Figure 1). However, the NMR spectra revealed that there were traces of oxygenated functional groups (Figure 2). The GPC analysis of the N-PEWs showed that they have a lower molecular mass than O-PEWs, which could make it a more accessible carbon source [16]. Although the hydrocarbon chains are hydrophobic, their shorter chains (after sonication [21]) would be able to be distributed within the TSB or BSM media, and from the growth curves in Figure 3, the addition of the wax made a difference (+1.8 log10 CFU mL^−1^ ) when it was added to BSM fermentations. The cell growth analysis also showed that N-PEWs do not have an adverse effect upon the growth rate of *C. necator* H16 in either TSB (not significant) or BSM. This was also reported by Radecka et al. [16] with O-PEW. In an experiment conducted by Zhang et al. [31] it was stated that PE has very low antimicrobial properties, limiting growth in Gram-positive and Gram-negative bacterial cells. However, in this study there was no evidence of antimicrobial activity. The low antimicrobial activity reported by Zhang et al. could have been due to the presence of antimicrobial moieties, such as chlorine or fluorine held within the polymer chains, rather than the PE itself. These contaminants could have been freed during the oxidation process and then dispersed into the medium to inhibit microbial growth. It is, therefore, likely that, in a similar way to O-PEW, N-PEWs are being successfully metabolized as fatty acids via *β*-oxidation. However, the GPC data was not able to fully support this theory as any reduction in Mn or Mw occurred in the controls without bacteria. Those smaller hydrocarbon chains that were not successfully recovered after the 48 h fermentation could have either been metabolized (in the inoculated flask) or they were fully dissolved in the medium. Another possibility is that some of the wax attached to the sides of the flasks as a thin film. For these reasons it was not possible to give a definitive value of the wax metabolized by the bacteria; a possible answer to this would be use a labelled variant of the wax. The presence of accessible carbon sources, such as fatty and carboxylic acids from O-PEW means that more carbon is present in the wax-supplemented cultures in a form that promotes the synthesis of PHAs [16]. It is possible that some of the larger alkane chains from N-PEWs that are more inaccessible for bacterial metabolism could be acting as oxygen inhibitors, limiting distribution within the system. This could then contribute to the stress conditions that push the growth culture towards the production of PHA [11,16]. 

The PHA yield of TSB cultures supplemented with N-PEW was 32% with a cell dry weight 1.42 g/L compared to shake flask experiments using TSB exclusively, which produced a lower yield of 20% and a dry weight of 0.98 g/L. There was growth of *C. necator* with N-PEW in BSM (shown by cell counts), however, no PHAs were recovered after 48 h. This is an indication that the addition of N-PEW into TSB has an effect upon the PHA production and the structure, with a Mn of 64,000 g/mol with a molecular distribution of 9.7 (Mw/Mn). The Mn results for PHAs produced with TSB only were lower, in the range of 52,000 g/mol with a distribution of 5.7 (Mw/Mn). These dispersity values indicate that N-PEW with TSB produces a more varied range of polyesters, and it is further evidence that N-PEWs are being metabolized.

^1^H-NMR was used to determine the structure of the copolymers produced. It was found that PHB was produced in the TSB only fermentation (data not shown) [16]. In the spectrum (Figure 4) the signals corresponded to the protons of the HB repeating units (labelled 1, 2, and 3) and additional signals at δ = 0.9 (-CH_2_CH_3_) and δ = 5.17 (-CH) correspond to HV repeated units that were observed. These signals are typical for PHAs with longer aliphatic groups in the *β*-position [32,33]. After integrating these signals, the content of monomer units other than 3-hydroxybutyrate (3-HB) has been estimated at 11 mol% 3-hydroxyvalerate (HV). However, due to some overlapping of certain signals, the protons of 2′ were not visible, although they have been reported in that location in PHBV previously [33]. 

In the N-PEW supplemented cultures, electrospray mass spectrometry (ESI-MS) determined copolymers were present. This method is known to provide more detail than GC and NMR when used for PHBV characterization. This is because it can corroborate whether a copolymer is a co-block polymer or randomly distributed. Therefore, it is feasible to find the precise sequence distribution of the oligomers generated and construct their sequences [34]. The oligomers that were obtained by partial depolymerisation with the controlled thermal degradation of the PHAs produced were induced by KHCO_3_ (Figure 5, Figure 6 and Figure 7). This procedure was conducted following the methodology of Kawalec et al. [30]. The E1cB degradation method displayed end groups with both unsaturated and carboxylic acid properties. Based on the mass assignment of single charged ions in the mass spectrum, the structure of the end groups and repeating units can be presumed to be sodium adducts of individual co-oligoester chains of 3-hydroxybutyrate (HB) and 3-hydroxyvalerate (HV). The fragmentation of these ions resulted from the random breakage of ester bonds along both sides of the oligomer chains and the results were consistent with previous studies [16,32,34]. The fragmentation data also suggests that the presence of N-PEW as a carbon source produces different PHAs to those synthesized from media supplemented with O-PEW, which has been reported to produce hydroxyhexanoate (HH) comonomer units, in addition to PHB and PHBV [16,32,33,34,35,36]. This is significant because an increase in the ratio of 3HV to 3HB can decrease the melting point and improve the mechanical strength of the polymers produced [37].

## 5. Conclusions

N-PEW, a substance produced from waste PE materials is a potential carbon source for PHA production with *C. necator*. The N-PEWs did not mix with growth media, such as TSB, due to their hydrophobic nature; however, with ultrasound sonication, these waxes formed an emulsion with reduced-length hydrocarbon chains that did provide an increased cell count, clearly acting as a viable carbon source. The structural and thermal characterization of the biopolymers produced showed that by adjusting the carbon source, the types of polymer synthesized can be changed. This variation affects the molecular masses and properties of the PHB or PHBV biopolmyers. N-PEW does not limit the growth rate of bacterial cells; in fact the cultures with N-PEW as an additional carbon source produced a higher yield of PHAs. BSM supplemented with N-PEW produced no PHAs, while TSB and N-PEW can produce yields of up to 40% (*w*/*w*), TSB only experiments gave a yield of 20% (*w*/*w*).

The ^1^H-NMR and ESI-MS/MS analysis indicated that in the presence of TSB/N-PEW the chains of the resulting biopolyester contained 3-HB repeating units with 11 mol% of 3-HV units randomly distributed along the copolymer chain. This is different to the PHAs reported from O-PEW/TSB fermentations, that produced PHBV and 3HH repeating units. The N-PEW/TSB media also yielded polymers with greater polymer diversity. Ultimately this analytical technique has enabled the characterization of PHAs synthesized from N-PEW/TSB media using *C. necator* H16, at the molecular level even when the contents of 3HB is high and the levels of other PHA oligomers (such as HV) are limited. This study demonstrates that plastic manufacturing companies could recycle more and utilize a wider range of materials for the production of value-added bioplastics and possibly direct the type of polyesters formed by selecting the level of wax oxidation. By using N-PEWs, food-based materials previously used for biopolymer synthesis, such as corn seed or sugar cane, could be reserved. These N-PEWs are a viable carbon source that can yield truly biodegradable, bio-compatible, non-toxic bioplastics for diverse applications.

## Figures and Tables

**Table 1:** The amount of PHA synthesized by *C. necator* using 4 g/L N-PEW. This experiment was conducted over a 48 h incubation period in TSB, TSB with N-PEW, and BSM with N-PEW at 30 °C (150 rpm).

Media	Average CDW (g/L)	Average PHA (g/L)	PHA (%*w*/*w*)
TSB only	0.98 ± 0.05	0.20 ± 0.05	20%
TSB with N-PEW	1.42 ± 0.20	0.46 ± 0.20	32%
BSM only	0.16 ± 0.06	ND	ND
BSM with N-PEW	0.20 ± 0.10	ND	ND

ND = non-detected; CDW = cellular dry weight.

**Table 2:** GPC results of unprocessed, pre-sonicated, post-sonicated, post-fermentation, and N-PEWs after shake flask exposure for 48 h without bacterial inoculation.

N-PEW Conditions	Number-Average Molar Mass (Mn) in g/mol	Molecular Mass Distribution Index (Mw/Mn)
Normal N-PEW (pre-sonication)	1600	3.5
Post-sonication N-PEWs	1200	1.2
Post-fermentation	1100	1.3
Post-shake flask (no bacteria)	1100	1.3
